# Giant Endometrial Polyp in a Postmenopausal Woman

**DOI:** 10.7759/cureus.12789

**Published:** 2021-01-19

**Authors:** Vivek Nair, Jitendra S Nigam, Jyotsna N Bharti, Biswajit Dey, Ashok Singh

**Affiliations:** 1 Pathology, Vikash Multispeciality Hospital, Sambalpur, IND; 2 Pathology/Lab Medicine, All India Institute of Medical Sciences, Patna, IND; 3 Pathology, All India Institute of Medical Sciences, Jodhpur, IND; 4 Pathology, North Eastern Indira Gandhi Regional Institute of Health and Medical Sciences, Shillong, IND; 5 Pathology, All India Institute of Medical Sciences, Rishikesh, IND

**Keywords:** phytoestrogens, estrogens, progesterone, aging

## Abstract

Endometrial polyps are the benign localized overgrowth of endometrial tissue composed of a variable amount of gland, fibroblast-like spindle cells stroma, and thick-walled blood vessels. They develop as a result of unbalanced estrogens and progestin. Polyps greater than 4 cm are considered giant polyps. We report a case of giant endometrial polyp in a postmenopausal woman who presented with postmenopausal bleeding without any history of hormone or drug intake. However, the possible cause may be the age and use of phytoestrogens in the daily routine diet for a long time.

## Introduction

Endometrial polyps (EPs) are the benign localized overgrowth of endometrial tissue protruding into the uterine cavity, affecting approximately 25% of women [1,2]. EP comprises a variable amount of gland, fibroblast-like spindle cells stroma, thick-walled blood vessels, and are lined by pseudostratified active or flat inactive epithelium [1,2]. The pathogenesis of EP is not well known; however, they are believed to develop due to unbalanced estrogens and progestin [1,2]. Phytoestrogens (PEs), a mimic of estrogen, are produced by plants and found abundantly in spices, herbs, and food [1]. Long-term consumption of PEs can act like estrogen, causing an unbalance between estrogen and progestin, leading to uterine pathologies such as endometrial hyperplasia or EP [1]. The use of tamoxifen and raloxifene is associated with an increased frequency of giant EPs [1]. EPs are usually less than 2 cm, and polyps greater than 4 cm are considered giant polyps [3]. We report the case of a giant EP in a postmenopausal woman who presented with postmenopausal bleeding without any history of hormone or drug intake.

## Case presentation

**Figure 1:**
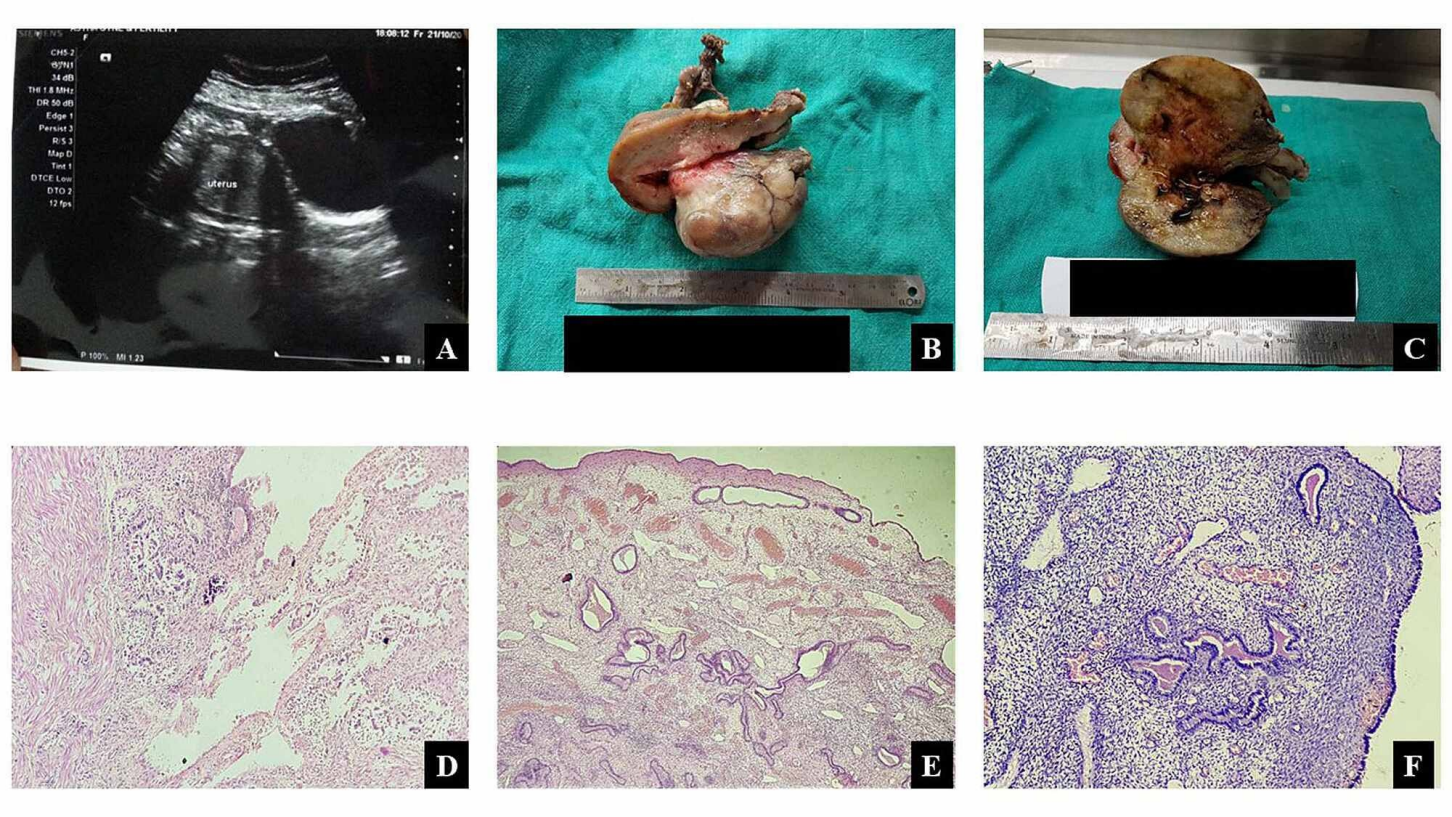
(A) The homogenous hyperechoic mass with a central anechoic area attached to the fundus occupying the uterine cavity. (B) A pedunculated polypoidal mass with a thin stalk attached to the fundus area. (C) Multiple small cystic spaces filled with altered blood. (D) Myometrium with endometrium and stalk of the EP. (E & F) Variable-sized glands with variable spindle cells stroma and many blood vessels. EP, endometrial polyp

A 67-year-old female, P4L4, presented in the gynecology outpatient department with a history of bleeding per vaginum for one month which was associated with spasmodic abdominal pain not relieved by medication. No history of prolonged illness, drug, or hormonal intake was elicited. The patient was of normal built for age with a body mass index of 20.1. Gynecological examination was unremarkable. Routine hematological and biochemical parameters were within the normal limits. On ultrasonography (USG), a homogenous hyperechoic mass measuring 48.4 × 38.2 mm with a central anechoic area attached to the fundus occupying the uterine cavity was detected. A diagnosis of submucosal fibroid was made on USG. Total abdominal hysterectomy was done, and the specimen was sent for histopathological examination. We received the total hysterectomy specimen. The uterus with cervix measured 11 × 7 × 5 cm with 1.5 cm long cervical canal. On cutting, a pedunculated polypoidal mass with a thin stalk attached to the fundus area, measuring 7 × 4 × 4 cm, was seen in the endometrial cavity. The external surface of the mass was smooth. The cut surface was grayish white with multiple small cystic spaces filled with altered blood. Microscopically, the cervix showed features of chronic non-specific cervicitis. The endometrium showed simple endometrial hyperplasia for age. The polyp showed variable-sized glands lined by cuboidal-to-columnar cells with variable spindle cells stroma and several blood vessels (Figure 1).

No atypia was noted in all the sections examined. A histopathological diagnosis of simple endometrial hyperplasia for age with benign hyperplastic EP was made. Further history was elicited from the patient for the possible etiology. She gave a history of daily intake of turmeric, garlic, ginger, onion, and the occasional intake of thyme and soybean in her diet, which are rich in PEs. Based on the clinical and histopathological findings, a final diagnosis of benign hyperplastic EP probably caused by the long-term dietary intake of PEs was made. The postoperative period was uneventful and the patient was doing well after six months of follow-up.

## Discussion

EPs are a common benign endometrial pathology that affects approximately 25% of women [2,4]. They are generally asymptomatic but can be seen with abnormal uterine bleeding in 13-50% of the cases. EPs may also present with infertility, premalignant, and malignant endometrial lesions [2]. In the present case, the patient presented with abnormal uterine bleeding associated with spasmodic abdominal pain without any history of prolonged illness, drug, or hormonal intake. The pathogenesis of EPs is not well known. However, by a systematic, semi-quantitative review on the pathogenesis of EP, Indraccolo et al. concluded that in postmenopausal women or during the first phase of the menstrual cycle, hyperactivation of β estrogen receptors on the α receptor enhance estrogen sensitivity in some areas of the endometrium leading to polyp formation [5]. Apoptosis via BCL-2 gene expression is blocked by estrogen-related inflammation, preventing their shedding during menstruation [5]. Indraccolo et al. also observed that there is a causative link between EP and BCL-2 expression, obesity, unbalanced estrogen therapy, imbalance between estrogen and progestins, estrogen-like effect, the relationship between estrogen and progestins, and tamoxifen, regardless of the timing [5]. Giant EPs are rare and are mostly associated with tamoxifen and raloxifene treatment [1]. Aging has also been linked to EPs [5]. In the present case, the patient was a 67-year-old postmenopausal female, and aging may be linked to the giant EP formation in the patient. The normal body mass index of the patient and normal biochemical results ruled out the possibility of any metabolic disorder. Certain plant-derived phytochemicals known as PEs are functionally and structurally similar to 17β-eestradiol (isoflavones) or synthetic estrogens such as diethylstilboestrol (lignins) [4]. Grains, fiber-rich foods, legumes, and nuts used in the diet rich in PEs present as glycosides [4]. Thyme, turmeric, garlic, ginger, onion, soy, pomegranate, licorice, red clover, hops, and Verbena are rich sources of PEs and phytoprogestins, which act as agonists and antagonists in vivo [6]. These are commonly consumed foods, herbs, and spices in an average Indian diet. Their activity in a female depends on their concentration, endogenous estrogen concentration, and menopausal status [1]. PEs with their estrogen-like effect can cause an imbalance with progesterone, which may lead to endometrial hyperplasia in postmenopausal women [1]. In the present case, there was a history of daily intake of turmeric, garlic, ginger, onion, and the occasional intake of thyme and soybean in her diet, which may be the possible cause of EP. Aging, obesity, arterial hypertension, postmenopausal period, and tamoxifen are also risk factors for malignancy development in the EP [2]. However, malignancy in EPs is uncommon and seen in 1-3% of the cases [2]. EPs larger than 15 mm are associated with endometrial hyperplasia, and in general, polyps measuring more than 10 mm are associated with an increased incidence of malignancy [2]. In the present case, no atypia or malignancy was seen after extensive sampling.

## Conclusions

The origin and pathogenesis of EPs are still not fully understood. However, in our case, we assume that aging and use of PEs in the daily routine diet for a long time may be the cause of the giant EP.
